# Genetic basis and principal component analysis in cotton (*Gossypium hirsutum* L.) grown under water deficit condition

**DOI:** 10.3389/fpls.2022.981369

**Published:** 2022-10-06

**Authors:** Aziz Ullah, Amir Shakeel, Hafiz Ghulam Muhu-Din Ahmed, Muhammad Naeem, Muhammad Ali, Adnan Noor Shah, Lichen Wang, Mariusz Jaremko, Nader R. Abdelsalam, Rehab Y. Ghareeb, Mohamed E. Hasan

**Affiliations:** ^1^Department of Plant Breeding and Genetics, University of Agriculture Faisalabad, Faisalabad, Pakistan; ^2^Department of Plant Breeding and Genetics, Faculty of Agriculture and Environment, The Islamia University of Bahawalpur, Bahawalpur, Pakistan; ^3^Institute of Agro-Industry and Environment, The Islamia University of Bahawalpur, Bahawalpur, Pakistan; ^4^Department of Agricultural Engineering, Khwaja Fareed University of Engineering and Information Technology, Rahim Yar Khan, Punjab, Pakistan; ^5^College of Life Science, Linyi University, Linyi, China; ^6^Smart-Health Initiative and Red Sea Research Center, Division of Biological and Environmental Sciences and Engineering, King Abdullah University of Science and Technology, Thuwal, Saudi Arabia; ^7^Agricultural Botany Department, Faculty of Agriculture (Saba Basha), Alexandria University, Alexandria, Egypt; ^8^Plant Protection and Biomolecular Diagnosis Department, Arid Lands Cultivation Research Institute, City of Scientific Research and Technological Applications, Alexandria, Egypt; ^9^Bioinformatics Department, Genetic Engineering and Biotechnology Research Institute, University of Sadat City, Sadat City, Egypt

**Keywords:** combining ability effects, gene action, line × tester, seed cotton yield, water deficit

## Abstract

Cotton is considered as the main crop in the agricultural sector of Pakistan. Water deficiency in this region in recent years has reduced the chances of high yields of cotton. Selection and creation of high-yielding varieties of cotton, even in water deficit conditions, is one of urgent tasks of today. For this purpose, 40 diverse genotypes of upland cotton were screened in normal and water deficit conditions in triplicate arrangement under split plot in a randomized complete block design. All the genotypes showed significant difference under both water regimes. Ten upland cotton accessions were screened out as water deficit tolerant (VH-144, IUB-212, MNH-886, VH-295, IR-3701, AA-802, NIAB-111, NS-121, FH-113, and FH-142) and five as water deficit sensitive (IR-3, CIM-443, FH-1000, MNH-147, and S-12) based on seed cotton yield and stress susceptibility index. These tolerant and sensitive genotypes were crossed in line × tester mating design. For further evaluation of genetic material, the seed of 50 F_1_ crosses and their 15 parents were field planted under normal and water deficit conditions during next cotton growing season. Traits related to yield under the study showed significant variations among the accessions and their half sibs. The results of the principal component analysis (PCA) exhibited that total variation exhibited by factors 1 and factor 2 were 55.55 and 41.95%, respectively. PCA transformed the variables into three factors, and only two factors (F1 and F2) had eigenvalue > 1. The degree of dominance revealed that all parameters were highly influenced by non-additive gene action under both water regimes. Furthermore, the line VH-295 and tester CIM-443 had better yield performance under water deficit stress. The cross-combinations, viz., VH-144 × S-12, NIAB-111 × IR-3, and VH-295 × MNH-147, were the best for yield contributing traits. These combinations may be helpful for germplasm enhancement on large scale under water scarcity. All the studied traits have non-additive types of gene action suggesting the usage of these genotypes in cotton hybrid development program against water deficit tolerance.

## Introduction

As global climate change continues, the decline in crop yields in agriculture will continue to increase as a result of water deficit. According to researches, by 2050, because of a 2–4°C increase in temperature and a sharp decrease in precipitation, 30% of the yield of agricultural crops appears to be lost (Ben-Asher et al., 2007). Pakistan's agriculture is both rain-fed and irrigated. In Pakistan, water availability during cotton growing season (kharif season 2021) recorded at 65.1 million-acre feet (MAF). During the monsoon season (July–September) 2021, rainfall recorded at 125.0 mm showing a decline of 11.3% against the normal average rainfall of 140.9 mm. During post-monsoon season (October–December) 2021, rainfall stood at 23.5 mm against the normal average rainfall of 26.4, showing a decrease of 11.2% (Anonymous, 2021-22). Cotton is a major source of fiber, food and feed in the world as well as in Pakistan. Pakistan is ranked fourth among the world's major cotton producing countries. It has a 0.6 % share in GDP and contributes 2.4 % in agriculture value addition (Anonymous, 2021-22), but, in current climatic change condition, the production of cotton varies greatly due to various biotic and abiotic stresses. Water deficit stress, among the various abiotic stresses, is an important factor that reduces the seed cotton yield (Haq et al., 2017).

Water is a key factor for plant growth, development, and yield attributes. Cotton plant is glycophytic in nature and shows medium tolerance to water deficit, as compared to other major crops. Unlike sorghum crop, which is grown in hot and dry climates, the cotton plant is generally not described as a resistant crop against water deficit (Ben-Asher et al., 2007); however, cotton has good adaptability in semi-arid regions (Malik et al., 2006; Amanov et al., 2020). Harsh climatic conditions badly affect the growth, quality, and yield of cotton crop (Iqbal et al., 2017).

In cotton, the critical stages which are highly responsive to water deficit are flowering and boll formation. The boll retention is highly reduced under severe water deficit conditions (Iqbal et al., 2018). A significant reduction in number of bolls per plant and yield of cotton plant was observed under water deficit conditions (Iqbal et al., 2017; Bakhsh et al., 2019). Abrupt water deficit episodes resulted in drastic yield reduction and poses threat for sustainable production in plants (Wang et al., 2016; Hussain et al., 2018). Timely irrigation is not only helpful for sustainable yield but also enhances stress tolerance capability of cotton plant (Zahoor et al., 2017; Farooq et al., 2019). Depending upon the severity and duration of stress, 50–70% yield losses were observed in cotton (Berry et al., 2014). Bolek (2007) and Shavkiev et al. (2019) stated in his experiments that the yield of plant is decreased by 39% due to water stress during flowering stage. Karademir et al. (2011) defined a decrease in seed cotton yield by 48.04% under water scarcity.

In severe water deficit conditions, synthesis and translocation of carbohydrates to reproductive parts of plant is reduced and depletion of reserved starch is fastened (Galmes et al., 2007; Abid et al., 2016). This phenomenon ultimately resulted in malnutrition of the plant reproductive organs due to which boll size and weight are decreased (Iqbal et al., 2017). Final impact of this malnutrition is dropping of leaves and fruits from plant, and final yield is drastically reduced (Pettigrew, 2004). The shedding of flowers and leaves due to water deficiency in cotton is also reported by many scientists (Bozorov et al., 2018; Shavkiev et al., 2020).

To cope with water deficit, better understanding of morpho-physiological mechanisms, *that is*, escapes, avoidance and tolerance, and their response to confer water deficit tolerance in plant is necessary. Since water deficit is the major environmental stress in agriculture worldwide, developing varieties having better yield under water deficit conditions is an important area in the plant breeding (Cattivelli et al., 2008). Two requirements are necessary, to develop or improve existing variety under water deficit conditions. First, variability must be present for water deficit tolerance in the crop plant, and second, the variation must have some genetic basis. Genetic variability among the genotypes is considered as key factor for plant breeders (Ul-Allah et al., 2019). Screening and selection of desirable parents in existing germplasm is the basic step to develop water deficit tolerant cotton genotypes. Different methods have been used to classify the several cotton genotypes by several scientists. According to Kim et al. (2013), among multivariate methods, principal component analysis (PCA) is a frequently used method to classify samples.

Previous research has indicated that the variability in water deficit stress tolerance in cotton crops is restricted, but a few studies have indicated that considerable amount of diversity against water deficit tolerance is also existing at maturity stage. The information about response of plant to water deficit is essential for improving the water deficit tolerance since morphological traits have been usually used to classify water deficit tolerant and sensitive genotypes in upland cotton (Jaleel et al., 2009; Raja et al., 2020). The main advantages of using these morphological traits in screening include no requirement of any specialized equipment for measuring them. Significant variation has been reported in various seed cotton yield-related traits (Mahmood et al., 2006; Ullah et al., 2019a).

It is challenging to breed genotypes/cultivars under water deficit stress due to a lack of quick screening tools. Creating repeatable water deficit conditions is difficult when huge amount of genotypes/lines are to be evaluated efficiently under water deficit conditions (Ramirez-Vallejo and Kelly, 1998). The various indices related to water deficit stress have wide application in screening huge germplasm in crop plants. The indices which have been associated with water deficit tolerance are calculated on the basis of yield loss under normal and water deficit conditions and provide information about tolerance to water deficit as reported by the researcher (Mitra, 2001). Stress susceptibility index (SSI) was introduced by Fischer and Maurer (1978) to screen huge genotypes. Guttieri et al. (2001) shown that stress susceptibility index less than a unit showed that genotype is a water deficit tolerant, while more than unit showed that genotype is sensitive to water deficit.

Previously, a lot of research work has been done regarding combining ability effects for various yield-related traits in cotton under normal irrigation. The glaring examples include Ahuja and Dhayal (2007), Ali and Awan (2009), and Simon et al. (2013). The research work related to the combining ability effects for various yield-related traits under water deficit condition is very important, but a very little work has been done on this aspect. However, some researchers (Soomro et al., 2012; Ullah et al., 2019b) have conducted study on combining ability regarding water deficit stress in cotton crop. Relative contribution of general and specific combining ability variances to the total phenotypic variance of population is very important in interpreting genetic structure of a breeding population and consequently in deciding the breeding methodology. High estimates of general combining ability variances indicate predominance of additive gene action, while high estimates of specific combining ability variances indicate predominance of non-additive and dominance gene action. Combining ability analysis of cultivars and their filial generation combinations is thus important to exploit the relevant type of gene action in the breeding program. For the estimation of the combining ability effects of parents and their crosses, the line × tester analysis is a good tool. Combining ability describes the breeding value of parental lines to produce hybrids. The beauty of this mating system is that there are no assumptions except the lines and testers should possess diverse genetic nature for the analysis. Sprague and Tatum (1942) stated that GCA effects are due to additive types of gene action, but SCA effects were due to genes which are non-additive (dominant or epistatic) types of action.

Some researchers (Sarwar et al., 2012 and Noor and Qayyum, 2020) have indicated non-additive type of gene action for number of bolls, boll weight, and seed cotton yield in cotton under water deficit conditions, while Shakoor et al. (2010) indicated additive type of gene action for number of bolls per plant in cotton under water deficit conditions. Scientists (Subhan et al., 2003) found high SCA than GCA for seed cotton yield, boll weight, and number of bolls per plant which showed dominance variance. The study of Raza et al. (2013) showed additive types of gene action for seed cotton yield and boll weight. Some scientists (Patel et al., 2007) reported that there was preponderance of non-additive genetic effects for seed cotton yield owing to higher SCA variance than GCA. The results of Shaukat et al. (2013) revealed that seed cotton yield exhibited higher SCA variances as compared to GCA, indicating non-additive genetic effects. The basic objective of this research was to study the inheritance pattern of gene action and combining ability of different yield-related attributes in cotton under water deficit conditions. This study will be helpful not only for choosing an appropriate breeding program, but also for selecting superior parents and F_1_'s, which can perform best under water deficit environment.

## Materials and methods

### Screening of germplasm under field condition (Experiment 1)

#### Experimental site, irrigation condition plant material, and experimental design

The 40 diverse cotton genotypes were collected from different research institutes, that is, NIAB Faisalabad, CCRI Multan, IUB Bahawalpur, and CRS-AARI Faisalabad. This experiment was carried out in the research area of Department of Plant Breeding and Genetics (PBG), University of Agriculture Faisalabad (UAF), Pakistan, during normal growing season of 2016. Faisalabad region has a semi-arid climate, according to the Koppen climate classification system (Khamdullaev et al., 2021). Hence, the region experiences very hot and humid summers and dry cool winters. The summer season starts in mid-April and continues until late October. May and June are the hottest months, while July, August, and the first half of September can be oppressively humid. June is the hottest month in Faisalabad. Climatic condition prevailing during present experimentation (April–November) in the year 2016 is given in Supplementary Figure 1 (Agromet Bulletin, Agriculture Meteorology Cell, Department of Crop Physiology, UAF, Pakistan).

In this experiment, cotton genotypes were field planted under two irrigation regimes, normal (To) and water deficit conditions (T_1_) under split plot in randomized complete block design repeated thrice during normal growing season of cotton on 10 May 2016. The main plots contained irrigations, while sub-plots contained genotypes in each replication. Ten plants of each genotype were grown in a single row. There were 75 cm and 30 cm distance between row to row and plant to plant, respectively. All recommended agronomic practices from sowing to maturity and at the time of harvesting were adopted.

Cotton is irrigated according to the 2-4-2 (pre-flowering– flowering–boll opening) sequence with two irrigations before flowering, four irrigations during flowering, and two irrigations during boll opening phases. This optimal irrigation protocol is widely used for cotton agriculture field in Pakistan. A modified irrigation protocol was also developed for deficient irrigation conditions. It has a 1-2-1 sequence, which limits 50% water availability during pre-flowering, flowering, and boll development stages as compared to normal irrigations (Kirda et al., 2005).

#### Data scoring

At the maturity stage, when drought symptom appeared, five plants of each genotype per replication and from each treatment were tagged for recording the data for seed cotton yield per plant (g). The open bolls were picked by three picks at maturity, seed cotton yield was weighed in grams, and then, average weight was calculated. The stress susceptibility index (SSI) was counted by using the following relationships (Fischer and Maurer, 1978).


$$\text{Stress susceptibility index }(\text{SSI})=1-(\text{Ys}/\text{Yp}))/(1-(\bar{\text{Y}}\text{s})/\bar{\text{Y}}\text{p}))$$

Yp and Ys indicated yield under normal and water deficit condition, while *Ȳ*s and *Ȳ*p indicated average yield in water deficit and normal conditions for all studied genotypes, respectively.

### Statistical analysis

Collected data were exposed to analysis of variance by using Statistix 8.1., and principal component analysis (PCA) was done on the mean data by using XLSTAT software (Khodadadi et al., 2011).

### Development of line × tester population

For the development of genetic material, 10 drought tolerant (VH-144, VH-295, IUB-212, IR-3701, MNH-886, AA-802, NS-121, NIAB-111, FH-113, and FH-142) and five drought sensitive (CIM-443, MNH-147, S-12, IR-3, and FH-1000) genotypes were sown in pots in the glasshouse of Department of Plant Breeding and Genetics, University of Agriculture, Faisalabad (Pakistan) during winter season 2016–2017 to produce F_1_ crosses. Ten (10) pots were assigned to each genotype, and six seeds per pot were planted to have three plants per pot after germination all the agronomic practices and plant protection measures were adopted from sowing to plant maturity. When the parents started flowering, these were crossed in line × tester mating design (Kempthorne, 1957). In the evening, suitable buds of the lines (female parent) were emasculated and covered with glycine bags to prevent the pollen contamination.

The sufficient amounts of pollens were collected from the tester plants in petri dish, and these pollens were dusted on the stigma of emasculated buds in the following morning. Numerous pollinations attempts ranging from 100 to 200 crosses for each cross-combinations were made to obtain sufficient amount of crossed seeds. Some buds from both male and female parents were also bagged to develop selfed seed.

At maturity, self-fertilized bolls from fifteen parents and crossed bolls from 50 F_1_ crosses of each combination (fully opened) were picked out by handpicking to get seed cotton. F_1_ seed was obtained after ginning with the help of single roller electric gin. Extreme attention was given to avoid the seeds of different genotypes from mixing during process of ginning. On an average, 08–15 seeds were obtained from a crossed boll. A total of 80–120 seeds were obtained from each cross-combination.

### Evaluation of genetic materials (Experiment 2)

#### Experimental site, irrigation condition, plant material and experimental design

This experiment was also carried out in the research area of Department of Plant Breeding and Genetics (PBG), University of Agriculture Faisalabad (UAF), Pakistan, during 2017. Climatic condition prevailing during present experimentation (April–November) in the year 2017 is given in Supplementary Figure 2 (Agromet Bulletin, Agriculture Meteorology Cell, Department of Crop Physiology, UAF, Pakistan). To examine the genetics of water deficit tolerance for various yield and yield-related attributes in cotton, 50 F_1_ crosses along with 15 parents (ten lines and five testers) were field planted under normal and water deficit conditions during normal cotton growing season on 14 May 2017. This experiment was conducted by using normal irrigations (To) and giving stress, 50% reduced irrigations (T1). This experiment was also carried out under split plot in randomized complete block design repeated thrice. The water levels were kept in main plot whereas genotypes in subplot. Seeds of each of the 65 entries per replication and treatment were planted in single row plot having ten plants each. There were 75 cm and 30 cm distance between row to row and plant to plant, respectively. All recommended agronomic practices from sowing to maturity and at the time of harvesting were adopted.

#### Data scoring

The five plants per replication and from each treatment for each genotype were tagged to score the data of boll weight (g), number of bolls per plant, seed cotton yield (g), and ginning out-turn%. The mature bolls were picked by three picks, and seed cotton for all the plants in three replications was collected in paper bags separately. The picking was done when the dew was evaporated. The seed cotton yield was weighed on electronic balance.

### Statistical analysis

The data noted were exposed to simple analysis of variance (Steel and Torrie, 1980). The traits that were found significant were analyzed for general and specific combining ability following line **×** tester analysis by Kempthorne (1957).

## Results and discussion

The genotypes showed significant differences for seed cotton yield. These were further analyzed by principal component analysis (PCA).

### Mean performance (Experiment 1)

The yield and water deficit tolerance being complex traits were affected by many factors. The mean values are displayed in Table 1. Regarding seed cotton under water deficit condition, the genotype such as FH-113 with highest mean value (97.70 g) exhibited water deficit tolerance followed by the genotypes IUB-212 (97.19 g) and NIAB-111 (85.74 g). The genotypes S-12, SB-149, CIM-443, and MNH-147 having lowest values of 16.82, 19.73, 23.22, and 23.9 g, respectively, were found sensitive to water deficit. The variable expressions of forty cotton genotypes for seed cotton yield under drought stress indicated that there was genotypic variability for drought tolerance. The presence of variability among genotypes for different traits under water-stressed conditions has been reported (Mvula et al., 2018).

**Table 1:** Mean values of forty cotton genotypes for seed cotton yield under normal (Yp), seed cotton yield under water deficit (Ys), and stress susceptibility index (SSI).

**Sr. No**.	**Genotype**	**Yp(g)**	**Ys(g)**	**SSI**
1	MNH-147	122.16	23.9	1.47
2	MNH-886	97.3	49.22	0.91
3	S-12	64.91	16.82	1.36
4	CRS-2007	125.45	49.71	1.29
5	SB-149	52.82	19.73	1.15
6	AA-703	76.94	30.65	1.1
7	MG-6	122	35.59	1.3
8	FH-118	130.07	53.94	1.21
9	MNH-888	51.73	28.66	0.82
10	CRS-456	144.72	34.28	1.4
11	FH-169	84.18	34.66	1.08
12	FH-113	137.39	97.7	0.41
13	CIM-443	94.98	23.22	1.38
14	FH-172	76.69	45.16	0.75
15	FH-175	94.35	47.36	0.91
16	IUB-212	138.73	97.19	0.81
17	FH-171	80.55	35.93	1.01
18	VH-295	57.06	45.83	0.36
19	NIAB-111	146.61	85.74	0.76
20	FH-941	126.51	32.82	0.89
21	FH-114	74.75	30.72	1.08
22	VH-282	113.14	38.93	1.2
23	IR-901	106.3	43.48	1.08
24	FH-170	147.84	78.43	0.99
25	NS-121	65.79	44.15	0.6
26	CIM-707	65.42	42.05	0.65
27	AA-802	104.71	54.6	0.67
28	IR-3	91.69	30.59	1.22
29	NIAB-820	73.94	40.63	0.83
30	AS-01	143.69	27.08	1.49
31	CIM-240	55.96	34.95	0.69
32	VH-148	153.65	77.48	0.91
33	NS-131	92.14	44.3	0.76
34	FH-1000	67.49	24.39	1.17
35	VH-144	85.39	61.71	0.51
36	FH-142	127.27	77.33	0.72
37	IUB-222	59.38	24.77	1.07
38	IR-3701	95.43	58.35	0.83
39	VH-283	57.74	32.45	0.8
40	KZ-181	137.71	37.02	1.34

For stress susceptibility index, VH-295 was the best performer with lowest (0.36) mean value followed by CRS-456 (0.40) and FH-113 (0.41) showing tolerance against drought stress. The genotype AS-01 was the poorest performer with highest value (1.49) followed by the genotypes MNH-147 (1.47), CRS-456 (1.4), CIM-443 (1.38), and S-12 (1.36). Among the different genotypes, VH-295, CRS-456, and FH-113 exhibited low stress susceptibility index as compared to other genotypes. Therefore, these with low stress susceptibility index (SSI) were selected as drought tolerant genotypes. Furthermore, these three genotypes showed higher tolerance due to the low stress susceptibility index. The stress susceptibility index (SSI) refers to the rate of change (for each genotype in yield between the normal and stress) relative to the mean change for all genotypes.

An SSI value of < 1 showed low sensitivity (good yield stability), and >1 showed high sensitivity to drought (poor yield stability). The genotypes AS-01, MNH-147, CIM-443, and S-12 showed sensitivity due to the high SSI value. Talebi et al. (2009) and Ullah et al. (2019a) by using stress susceptibility index (SSI) evaluated tolerance to drought in many genotypes of cotton and wheat and identified variation regarding SSI from year to year. The effectiveness of selection indices based on the stress severity supports that various conditions of stress affect the crop yield as reported by the researcher (Ullah et al., 2019a).

### Principal component analysis

A principal component analysis (PCA) was performed, and after that, genotypes were exposed to biplot analysis to see the association among them.

**Figure 1:**
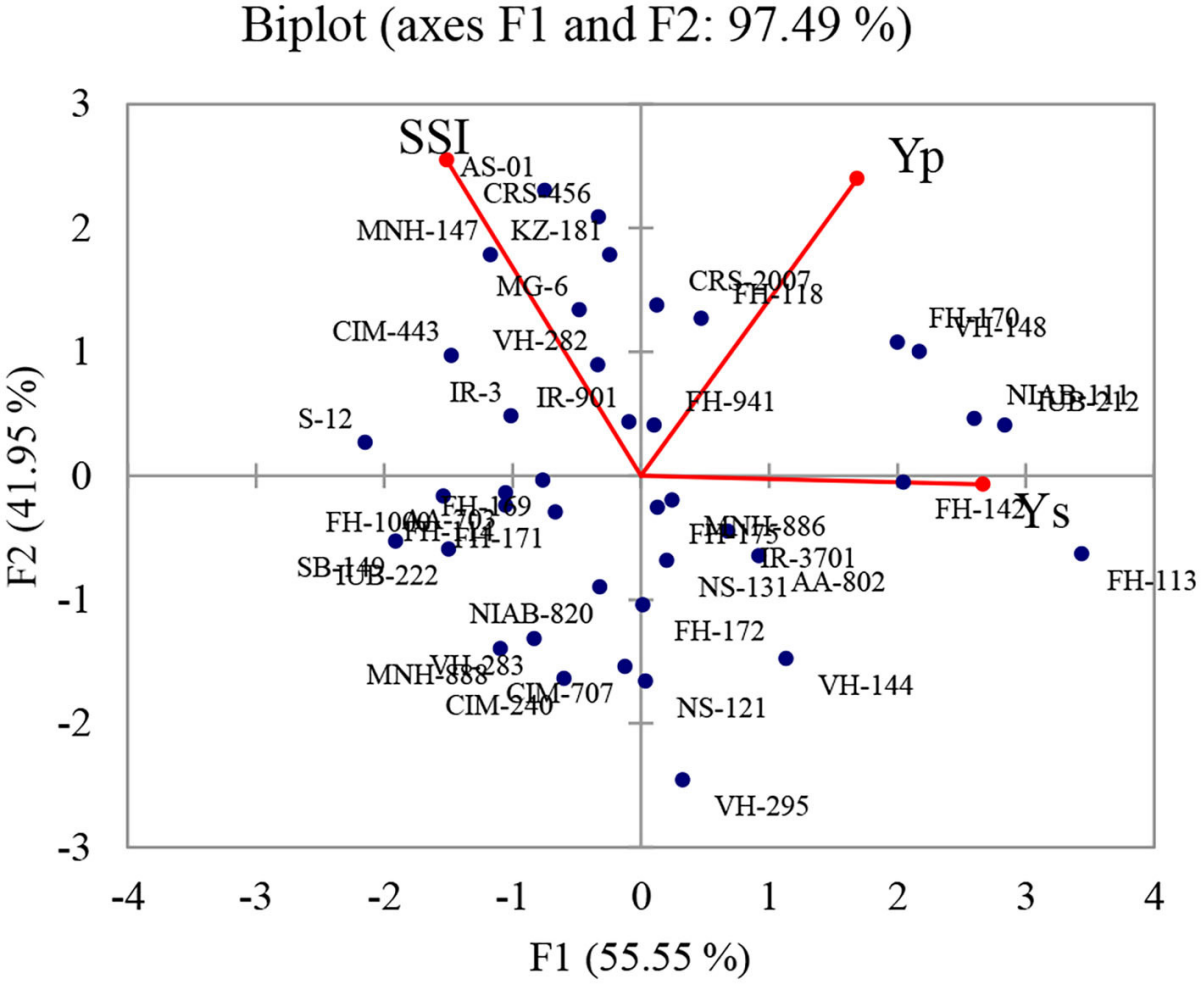
Biplot for water deficit tolerance in seed cotton yield and stress susceptibility index in 40 diverse cotton genotypes based on first two components. Seed cotton yield under normal (Yp), seed cotton yield under drought (Ys), and stress susceptibility index (SSI).

Many researchers have used biplot analysis for comparing genotypes for different criteria. According to Ullah et al. (2019a), data were considered in each component with Eigen F value >1 which determined at least 10% of the variation. The higher eigenvalues were considered as best representative of system attributes in principal components. Only two components (PCs) showed more than 1 eigenvalue and exhibited about 97.49% cumulative variability (Figure 1); therefore, these two PCs were used for further explanation (Table 2).

**Table 2:** Eigenvalue, variability, and cumulative variability of different factors; correlations between variables and factors; contribution of the variables (%) in variability of different factors; based on principal component analysis.

**Variables**	**Factor 1**	**Factor 2**
Eigenvalue	1.666	1.258
Variability (%)	55.548	41.947
Cumulative Percentage	55.548	97.494
Correlations between variables and factors		
Yp	0.622	0.769
Ys	0.984	−0.022
SSI	−0.558	0.817
Contribution of the variables (%) in variability of different factors		
Yp	23.211	46.946
Ys	58.09	0.04
SSI	18.699	53.014

Yp, seed cotton yield under normal condition; Ys, seed cotton yield under water deficit condition; SSI, stress susceptibility index.

Chunthaburee et al. (2016) have already explained the only first principal component (first PC) and second principal component (second PC) which have major contribution in their correlation study as other PCs cover only little information of data sets.

First two factors (F_1_ and F_2_) contributed 55.55 and 97.49% cumulative variability, respectively (Table 2). Different variables had different percentage of contribution in total variability. Seed cotton yield under normal condition contributed 23.21 and 46.95% variability in 1 st and 2 nd factor, respectively (Table 2). Seed cotton yield under water deficit condition had very low percent contribution for F_2_, but for F_1_ this had contributed 58.09% variability. Stress susceptibility index contributed 18.70% variability of F_1_ and 53.01% variability of F_2_, respectively (Table 2). The studies of Ullah et al. (2019a) seemed to come to an agreement with the present investigation.

Regarding yield under stress condition, highest estimates were recorded in NIAB-111, IUB-212, FH-114, FH-113, VH-148, and FH-170 representing high water deficit tolerance. IR-3701, AA-802, VH-144, and MNH-886 possessing positive side of the biplot also showed higher estimates. Minimum estimates were observed in CIM-443, S-12, MNH-147, and FH-1000 revealing drought sensitivity. In addition, IR-3, FH-169, AA-703, FH-171, and FH-114 were also sensitive to drought conditions.

The minimum estimates of SSI were recorded in VH-295, VH-144, NS-121, and FH-172. This index value was also low in AA-802, IR-3701NS-131, and MNH-886 which were present on negative side of biplot which were also considered as water deficit tolerant. The maximum estimates of SSI were observed in AS-01, CRS-456, CIM-443, and KZ-181 representing water deficit sensitivity. In addition, MG-6, VH-282, IR-3, and IR-901 were also sensitive to water deficit condition. Stress susceptibility index (SSI) showed significant negative association with yield under water deficit conditions (Ys). The genotypes with high SSI values revealed higher seed cotton yield under normal conditions, and on the contrary, there was a trend with low SSI scores to be related with higher yield under water deficit conditions. Similar type of trend was found by Sio-Se Mardeh et al. (2006), Talebi et al. (2009), and Karimizadeh et al. (2011). The overall performance on the basis of both Ys and SSI, and the genotypes NIAB-111, IUB-212, FH-142, FH-113, VH-295, VH-144, NS-121, AA-802, IR-3701, and MNH-886 (water deficit tolerant) were better performer, and MNH-147, CIM-443, S-12, IR-3, and FH-1000 (water deficit sensitive) were poor performer.

The results of the principal component analysis (PCA) exhibited that total variation exhibited by factors 1 and factor 2 were 55.55 and 41.95%, respectively. PC1 was positively associated with both yield under normal and stress condition. This component was associated with water deficit tolerance (yield potential). Varieties having high PC1 value are considered good yielder under normal and water deficit stress. These findings are in accordance to the findings of Golabadi et al. (2006) in wheat (durum).

The PC2 showed 41.95% of the variation (total yield variation) and correlated with Yp and SSI. PC2 is associated with stress sensitivity. Keeping in mind the tolerant and sensitivity, PC1 and PC2 were known as “yield potential and stress sensitivity,” respectively. The results showed that FH-113, FH-142, NIAB-111, MNH-886, IR-3701, VH-144, NS-121, and AA-802 were closely located to the best drought tolerance parameters with high PC1 as compare to PC2 values (Figure 1). The most of genotypes with lower PC1 and higher PC2 values were found as sensitive genotypes. These included CRS-456, KZ-181, MG-6, MNH-147, IR-3, S-12, and FH-1000. These findings are similar to Kaya et al. (2002) who found that varieties with higher PC2 and lower PC1 scores had good yields and varieties with lower PC2 and higher PC1 value had low yield.

Comparison of 40 genotypes (varieties/lines) shown valuable information about potential of the material to withstand water deficit stress and allowed the identification of some water deficit tolerant and sensitive genotypes. Comparison of genotypes based on seed cotton yield and stress susceptibility index suggests that they might be important genetic source of genes for enhancing water deficit tolerance. In previous study regarding water deficit tolerance in cotton, Ullah et al. (2008) and Ullah et al. (2019a) showed the great variation in studied material under normal and water deficit conditions and thus support the present investigation.

### Combing ability of parents and their crosses

Line × tester analysis of variance for each yield-related trait was conducted separately under both normal and water deficit conditions. Mean squares were differed significantly for all traits (Table 3).

**Table 3:** Mean square values of line × tester analysis for various traits under normal and water deficit condition.

**SOV**	**DF**	**Normal condition**				**Water deficit Condition**			
		**BP**	**BW**	**SCY**	**GOT**	**BP**	**BW**	**SCY**	**GOT**
Rep.	2	7.88^**^	0.01^**^	93.44^**^	4.13^**^	17.27^**^	0.11^**^	515.36^**^	5.64^**^
Gen.	64	89.73^**^	1.99^**^	3,412.19^**^	23.79^**^	58.82^**^	1.42^**^	1,760.91^**^	20.42^**^
Parents	14	113.73^**^	3.08^**^	5,115.42^**^	24.20^**^	99.01^**^	0.89^**^	1,515.41^**^	27.32^**^
Crosses	49	84.69^**^	1.66^**^	2,969.51^**^	23.73^**^	48.35^**^	1.52^**^	1,787.98^**^	16.90^**^
P. vs Crosses	1	0.64	3.11^**^	1,258.47^**^	21.11^**^	9.11^**^	3.66^**^	3,871.34^**^	96.17^**^
Lines	9	76.86	1.85^**^	4,175.95^**^	7.86^**^	54.30^**^	1.48^**^	1,403.15^**^	13.13^**^
Testers	4	173.46	3.59^**^	7,059.79^**^	227.04^**^	144.06^**^	6.31^**^	7,577.82^**^	33.39^**^
L x T	36	76.79^**^	1.40^**^	2,213.43^**^	5.11^**^	36.23^**^	1.00^**^	1,240.88^**^	16.01^**^
Error	128	1.17	0.01	25.43	0.97	1.01	0.01	17.65	0.86

*Significant;

** highly significant; Df, degree of freedom; Rep, replications; Gen, genotypes; BP, number of bolls per plant; BW, boll weight; SCY, seed cotton yield; GOT, ginning out-turn%.

#### Number of bolls per plant

The general combining ability (GCA) effects for number of bolls per plant in normal condition showed that maximum positive significant values were exhibited by VH-144 (5.15) which indicated that this line was good general combiner followed by VH-295 (1.47) and IR-3701 (1.03). In water deficit condition, among the lines, IUB-212 exhibited the maximum positive GCA effects for this trait followed by VH-144 (4.25 and 1.82, respectively) while FH-142 presented maximum negative GCA estimates (−2.56) for this trait. Among testers, maximum GCA estimates were exhibited by CIM-443 followed by IR-3 (2.53 and 1.39, respectively) (Table 4). General combining ability effects are equivalent to additive effects, which are important genetic information to find out the desirable general combiner for improving traits of interest (Wu et al., 2010). When GCA of parental genotypes was compared with each other, FH-142 and CIM-443 cultivars maintained their best combinatory feature by number of bolls per plant under normal and water deficit conditions. These genotypes may be used in breeding program for the improvement of high yield having enhanced water deficit tolerance in upland cotton.

**Table 4:** Estimation of general combining ability effects for various yield-related traits under normal and water deficit condition.

	**Normal condition**				**Water deficit condition**			
**Parents/Traits**	**BP**	**BW**	**SCY**	**GOT**	**BP**	**BW**	**SCY**	**GOT**
VH-144	5.15^**^	0.40^**^	32.36^**^	−0.41	1.82^**^	0.07^**^	7.04^**^	−0.51^*^
IUB-212	0.43	0.56^**^	16.72^**^	−0.80^**^	4.25^**^	−0.29^**^	7.27^**^	−0.39
MNH-886	−1.80^**^	0.37^**^	3.97^**^	−0.59^*^	−1.02^**^	0.02	−4.51^**^	−0.86^**^
VH-295	1.47^**^	0.05	7.97^**^	−0.71^**^	0.74^**^	0.20^**^	9.31^**^	−0.08
IR-3701	1.03^**^	−0.01	2.91^*^	0	−0.38	−0.42^**^	−11.44^**^	0.3
AA-802	0.1	−0.07^**^	0.09	0.82^**^	−1.03^**^	−0.51^**^	−17.07^**^	1.21^**^
NIAB-111	−1.22^**^	−0.15^**^	−10.54^**^	−0.18	−1.32^**^	0.26^**^	1.7	0.38
NS-121	−0.63^*^	−0.39^**^	−14.65^**^	0.07	−0.16	0.24^**^	6.08^**^	0.19
FH-113	−1.62^**^	−0.24^**^	−16.95^**^	0.31	−0.33	0.44^**^	10.01^**^	−1.70^**^
FH-142	−2.90^**^	−0.51^**^	−21.87^**^	1.50^**^	−2.56^**^	−0.01	−8.38^**^	1.45^**^
S.E	0.28	0.03	1.3	0.25	0.26	0.02	1.08	0.24
IR-3	0.87^**^	0.21^**^	9.06^**^	1.93^**^	1.39^**^	−0.74^**^	−15.78^**^	1.45^**^
CIM-443	3.54^**^	0.13^**^	15.48^**^	1.04^**^	2.53^**^	0.46^**^	22.79^**^	0.08
FH-1000	−0.22	0.16^**^	2.94^**^	0.50^**^	0.64^**^	0.29^**^	9.92^**^	−0.09
MNH-147	−1.36^**^	0.13^**^	−2.94^**^	1.36^**^	−2.42^**^	0.02	−8.51^**^	−1.53^**^
S-12	−2.82^**^	−0.62	−24.54^**^	−4.83^**^	−2.15^**^	−0.04^**^	−8.42^**^	0.09
S.E	0.2	0.02	0.92	0.18	0.18	0.01	0.77	0.17

* Significant;

** highly significant; BP, number of bolls per plant; BW, boll weight; SCY, seed cotton yield; GOT, ginning out-turn%.

The SCA results showed that FH-142 × CIM-443 exhibited the maximum SCA estimates (8.81), which indicated as good combination for improving this trait followed by FH-113 × CIM-443 and IUB-212 × S-12 (7.57 and 7.14, respectively). Under water deficit condition (Table 5), the maximum value of positive SCA estimates (4.65) was exhibited by VH-295 × CIM-443 followed by the cross VH-295 × MNH-147 (4.54), while maximum negative SCA value (−10.09) was estimated for the cross VH-144 × CIM-443 followed by VH-295 × IR-3 (−5.76) (Table 5). These findings are in accordance with Khamdullaev et al. (2021) who studied the productivity and productivity related traits, that is, number of boll per plant and boll weight in upland cotton under water deficit condition.

**Table 5:** Specific combining ability effects of crosses for various yield–related traits under normal (N) and water deficit (D) condition.

**Trait**	**BP**		**BW**		**SCY**		**GOT**	
**Cross**	**SCA(N)**	**SCA(D)**	**SCA(N)**	**SCA(D)**	**SCA(N)**	**SCA(D)**	**SCA(N)**	**SCA(D)**
VH-144 × IR-3	3.18^**^	3.94^**^	−0.33^**^	−0.68^**^	3.59	−9.69^**^	1.24^*^	0.82
VH-144 × CIM-443	−6.50^**^	−10.09^**^	−0.20^**^	−0.33^**^	−30.53^**^	−47.11^**^	0.51	−0.79
VH-144 × FH-1000	−0.49	0.31	−0.37^**^	0.58^**^	−12.99^**^	20.20^**^	−1.28^*^	−2.33^**^
VH-144 × MNH-147	−2.20^**^	2.41^**^	−0.01	−0.05	−8.60^*^	9.08^**^	−0.77	−0.97
VH-144 × S-12	6.01^**^	3.43^**^	0.90^**^	0.48^**^	48.54^**^	27.52^**^	0.30	3.25^**^
IUB-212 × IR-3	−2.30^**^	3.32^**^	−0.06	−0.42^**^	−11.24^**^	−9.22^**^	1.37^*^	2.24^**^
IUB-212 × CIM-443	−6.97^**^	−0.19	−0.15^*^	0.40^**^	−31.07^**^	12.52^**^	−0.03	−0.82
IUB-212 × FH-1000	1.54^*^	1.30^*^	0.59^**^	0.27^**^	25.65^**^	14.35^**^	−1.79^**^	−2.98^**^
IUB-212 × MNH-147	0.59	−8.23^**^	−0.44^**^	−0.50^**^	−9.06^**^	−37.44^**^	−0.67	0.78
IUB-212 × S-12	7.14^**^	3.80^**^	0.07	0.24^**^	25.71^**^	19.80^**^	1.12^*^	0.78
MNH-886 × IR-3	−1.20^*^	0.05	0.22^**^	−0.92^**^	−1.46	−21.31^**^	−0.91	0.98
MNH-886 × CIM-443	3.94^**^	−0.58	0.34^**^	−0.05	29.62^**^	−3.45	0.92	1.28^*^
MNH-886 × FH-1000	3.64^**^	3.53^**^	−0.11	−0.18^**^	10.84^**^	10.21^**^	−0.32	−0.45
MNH-886 × MNH-147	−0.79	−0.36	−0.26^**^	0.18^**^	−11.59^**^	3.82	−0.50	0.99
MNH-886 × S-12	−5.60^**^	−2.64^**^	−0.17^**^	0.97^**^	−27.41^**^	10.72^**^	0.81	−2.80^**^
VH-295 × IR-3	3.63^**^	−5.76^**^	0.03	−0.68^**^	14.89^**^	−34.07^**^	−0.11	0.19
VH-295 × CIM-443	1.10	4.65^**^	0.05	−0.01	6.92^*^	19.07^**^	−0.50	−0.85^*^
VH-295 × FH-1000	−3.67^**^	−0.78	0.10	−0.41^**^	−13.27^**^	−14.74^**^	−0.40	4.32^**^
VH-295 × MNH-147	2.70^**^	4.54^**^	0.34^**^	0.31^**^	20.79^**^	24.83^**^	−0.28	0.63
VH-295 × S-12	−3.76^**^	−2.65^**^	−0.52^**^	0.79^**^	−29.33^**^	4.91^*^	1.29^*^	−4.29^**^
IR-3701 × IR-3	6.73^**^	−1.97^**^	−0.19^**^	−0.38^**^	20.71^**^	−14.75^**^	0.29	−2.80^**^
IR-3701 × CIM-443	−2.67^**^	0.15	0.21^**^	0.52^**^	−1.97	12.80^**^	0.03	−1.77^**^
IR-3701 × FH-1000	−4.86^**^	3.01^**^	0.56^**^	−0.16^**^	−5.33	4.96^*^	0.60	1.10^*^
IR-3701 × MNH-147	3.48^**^	0.03	−0.41^**^	0.54^**^	2.21	12.81^**^	1.15^*^	0.77
IR-3701 × S-12	−2.68^**^	−1.23^*^	−0.18^**^	−0.52^**^	−15.62^**^	−15.81^**^	−2.08^**^	2.69^**^
AA-802 × IR-3	1.83^**^	−0.69	1.16^**^	−0.17^**^	40.82^**^	−4.89^*^	−1.98^**^	−0.42
AA-802 × CIM-443	−3.10^**^	−1.23^*^	0.53^**^	0.07	2.37	−4.62	0.02	−1.28^*^
AA-802 × FH-1000	2.49^**^	−2.68^**^	−0.90^**^	0.49^**^	−20.00^**^	1.88	1.80^**^	1.34^*^
AA-802 × MNH-147	3.37^**^	3.41^**^	0.04	−0.16^**^	13.12^**^	8.35^**^	0.76	2.30^**^
AA-802 × S-12	−4.59^**^	1.19^*^	−0.82^**^	−0.23^**^	−36.31^**^	−0.72	−0.60	−1.94^**^
NIAB-111 × IR-3	2.46^**^	2.91^**^	−0.84^**^	0.77^**^	−15.21^**^	33.33^**^	−1.43^*^	−1.92^**^
NIAB-111 × CIM-443	0.38	3.37^**^	1.14^**^	−0.30^**^	39.34^**^	5.01^*^	1.31^*^	1.04
NIAB-111 × FH-1000	−1.71^**^	−2.32^**^	−0.80^**^	−0.12^**^	−25.24^**^	−12.92^**^	−2.19^**^	−3.14^**^
NIAB-111 × MNH-147	−7.89^**^	−3.29^**^	0.54^**^	0.15^**^	−18.58^**^	−10.90^**^	1.09	3.40^**^
NIAB-111 × S-12	6.75^**^	−0.67	−0.04	−0.50^**^	19.69^**^	−14.52^**^	1.23^*^	0.62
NS-121 × IR-3	−6.19^**^	−2.33^**^	−0.12^*^	0.63^**^	−25.01^**^	8.95^**^	0.87	−0.77
NS-121 × CIM-443	−2.56^**^	1.19^*^	−0.61^**^	0.25^**^	−25.59^**^	12.12^**^	1.42^*^	0.99
NS-121 × FH-1000	−1.03	−1.71^**^	0.46^**^	−0.48^**^	9.54^**^	−19.17^**^	0.71	−1.18^*^
NS-121 × MNH-147	−1.03	−1.71^**^	0.46^**^	−0.48^**^	9.54^**^	−19.17	0.71	−1.18^*^
NS-121 × S-12	5.27^**^	0.18	0.72^**^	0.04	36.72^**^	0.25^**^	−2.13^**^	1.77^**^
FH-113 × IR-3	−4.45^**^	−0.61	1.16^**^	0.66^**^	13.05^**^	16.76	0.28	1.76^**^
FH-113 × CIM-443	7.57^**^	1.19^*^	−1.49^**^	−0.33^**^	−23.28^**^	−3.89	−1.85^**^	−0.24
FH-113 × FH-1000	−0.32	−0.11	−0.53^**^	−0.16^**^	−11.38^**^	−4.44^**^	1.46^*^	2.62^**^
FH-113 × MNH-147	1.22	−1.15^*^	0.62^**^	−0.21^**^	25.85^**^	−10.57	−0.27	−3.56^**^
FH-113 × S-12	−4.02^**^	0.68	0.24^**^	0.03	−4.24	2.14	0.39	−0.58
FH-142 × IR-3	−3.70^**^	1.12^*^	−1.02^**^	1.18^**^	−40.14^**^	34.89^**^	0.38	−0.09
FH-142 × CIM-443	8.81^**^	1.54^**^	0.18^**^	−0.21^**^	34.18^**^	−2.44	−1.82^**^	2.43^**^
FH-142 × FH-1000	4.41^**^	−0.55	1.00^**^	0.16^**^	42.18^**^	−0.33	1.40^*^	0.70
FH-142 × MNH-147	−4.99^**^	−0.02	0.03	0.17^**^	−18.47^**^	2.17	0.37	−3.54^**^
FH-142 × S-12	−4.53^**^	−2.10^**^	−0.19^**^	−1.30^**^	−17.76^**^	−34.29^**^	−0.33	0.50
S.E	0.62	0.58	0.06	0.04	2.91	2.43	0.57	0.54

* Significant;

** highly significant; BP, number of bolls per plant; BW, boll weight; SCY, seed cotton yield; GOT, ginning out-turn%.

#### Boll weight

Under normal condition, IUB-212 exhibited the maximum significant and positive value (0.56). Under water deficit condition, maximum significant positive GCA estimates were exhibited by FH-113 (0.44) and marked as best general combiner for the trait under study. The maximum value of negative GCA estimates was given by AA-802 followed by IR-3701 with a GCA value of −0.51 and −0.42, respectively, and hence marked as poor combiner. Regarding male parents (testers), CIM-443 exhibited significant and positive GCA estimate (0.46) followed by FH-1000 (0.29) (Table 4). Such kind of data has been obtained in the previous experiments (Wu et al., 2010; Ullah et al., 2019b).

Under normal condition, the results revealed that out of 50 crosses, 19 crosses exhibited significant and positive SCA estimates whereas 21 crosses showed significant and negative SCA effects. The crosses such as AA-802 × IR-3 and FH-113 × IR-3 were the best combinations because of maximum SCA estimates of equal value (1.16) followed by NIAB-111 × CIM-443 (1.14). Under water deficit condition, the highest positive value was shown by FH-142 × IR-3 (1.18) followed by MNH-886 × S-12 (0.97) showing as desirable combinations. FH-142 × IR-3 displayed as best cross-combination for boll weight, but the cross was originated from the parental line with poor GCA effects. Thus, the present study clarified that it is possible for parental lines with poor GCA effects to produce good new cross-combination. Comparable findings were given by Sajjad et al. (2016) and Khamdullaev et al. (2021).

#### Seed cotton yield

GCA effects for seed cotton yield in normal condition indicated that VH-144 showed the maximum significant and positive value (32.36) and was considered as best general combiner. VH-144, which was best general combiner for number of bolls and boll weight, also exhibited maximum value of GCA for seed cotton yield. GCA effects for seed cotton yield under water deficit conditions showed that maximum significant positive GCA estimates were exhibited by FH-113 (10.01), indicating its good combining ability for the trait under study. Among testers (male parents), CIM-443 exhibited significant and positive GCA estimates (22.79) followed by FH-1000 (9.92) (Table 4). The involvement of one of parent having high GCA would tend to increase the frequency of favorable alleles. Most of crosses with good SCA effects may be either due to good GCA of parents, indicating the preponderance of additive genetic effects as reported by the researcher (Kenga et al., 2004).

Under normal condition, the results revealed that out of 50 crosses, 20 crosses presented significant and positive SCA effects, other 23 crosses showed significant and negative SCA effect, whereas the remaining crosses exhibited non-significant results for this trait. The cross VH-144 × S-12 was considered as the best combination because of maximum SCA estimates (48.54) followed by the cross FH-142 × FH-1000 (42.18). Under water deficit condition, the highest significant and positive SCA estimates were exhibited by FH-142 × IR-3 (34.89) followed by NIAB-111 × IR-3 (33.33) indicating better performance of these cross-combinations. Present findings are in accordance with Wu et al. (2010) and Usharani et al. (2016). The cross-combinations VH-144 × CIM-443 and IUB-212 × MNH-147 exhibited poor performance with significant and negative SCA estimates (−47.11 and −37.44, respectively) for this trait. It should be noted that by seed cotton yield, the combination FH-142 × IR-3, obtained from FH-142 and IR-3 lines with negative GCA effect, has showed high SCA effect in water deficit condition. This indicates significant role of non-additive genes action. High SCA effects due to parents with low GCA revealed the influence of non-additive genetic effects and warn the researcher to avoid selection in early generations (Saidaiah et al., 2010).

#### Ginning out-turn %

In case of ginning out-turn %, positive GCA effects are desirable. Regarding this trait, among lines, FH-142 (1.45) and AA-802 (1.21) displayed positive and highly significant estimates under water deficit condition while FH-113 (−1.70), MNH-886 (−0.86), and VH-144 (−0.51) showed significant negative value. Among testers, IR-3 (1.45) presented positive significant values under water deficit condition (Table 4). The best combination was considered as AA-802 × FH-1000 with maximum significant and positive SCA estimates (1.80) followed by the cross FH-113 × FH-1000 (1.46) under normal condition.

In water deficit conditions, the highest significant and positive SCA effects were displayed by the VH-295 × FH-1000 (4.32) marked as the best combination followed by NIAB-111 × MNH-147 (3.40). Significant and negative SCA estimates were given by 14 crosses containing VH-295 × S-12 with highest value (−4.29) followed by FH-113 × MNH-147 (−3.56) and FH-142 × MNH-147 (−3.54) which were considered as the poor combination for this trait (Table 5). Positive SCA effects were observed in VH-295 × FH-1000 and NIAB-111 × MNH-147 crosses obtained from parental genotypes with high GCA effect by lint percentage trait. Similar data were obtained in the previous studies too (Adnan et al., 2006). The parents having high SCA effects indicated the role of dominant effects that allow the opportunity to the breeder for the development of hybrids or hybrid seed production program (Ali et al., 2013; Khan, 2014).

The crosses VH-144 × S-12, NIAB-111 × IR-3, and VH-295 × MNH-147 performed better for most of yield-related traits under water deficit condition with high-specific combining ability effects. Theses crosses can be used in variety of development program for drought pruned areas of Pakistan. Variance due to specific combining ability was greater for all traits showing non-additive gene effects under normal and drought stress. The investigation of Neelima et al. (2004) and Shakeel et al. (2001) is in accordance with the present investigations. Parents showing poor general combining ability but their cross-combination FH-142 × IR-3 showed good specific combining ability for seed cotton yield. Some cases involving good × good, good × poor, and poor × poor parents have been reported which result in hybrids with out-standing performance (Karademir et al., 2009; Imran et al., 2015). The variations in genetic make-up and different environmental conditions play important role in performance of parents and their cross-combinations (Pettersen et al., 2006).

### Genetic components

Combining ability effects relatively provides appropriate understanding on the genetic control of various plant characters. GCA to SCA ratio revealed predominance of non-additive type of gene action for number of boll per plant, boll weight, seed cotton yield, and lint percentage under normal and water deficit conditions (Table 6). Present findings are in accordance with Wu et al. (2010), Usharani et al. (2016), and Ullah et al. (2019b). Contrary to the findings of the present study, Khan et al. (2015) reported additive type of gene action while Jatoi et al. (2010) and Patel et al. (2014) reported both additive and non-additive type of gene action for traits under study.

**Table 6:** Estimation of genetic components of variation under normal and drought condition.

**Traits**	**Normal condition**		**Water deficit condition**	
	**∂ GCA**	**∂ SCA**	**∂ GCA**	**∂ SCA**
BP	0.105	25.207	0.16	11.74
BW	0.003	0.462	0.007	0.333
SCY	10	729.332	7.236	407.742
GOT	0.246	1.381	0.012	5.05

∂ GCA, estimate of GCA variance; ∂ SCA, estimate of SCA variance; BP, number of bolls per plant; BW, boll weight; SCY, seed cotton yield; GOT, ginning out-turn%.

Our findings are also supported by some other researchers (Sarwar et al., 2012 and Noor and Qayyum, 2020) who indicated non-additive type of gene action for number of bolls, boll weight, and seed cotton yield in cotton under water deficit conditions. Shakoor et al. (2010) indicated additive type of gene action for number of bolls per plant in cotton under water deficit conditions; these findings are contradictory to our findings. All traits showed significance of non-additive effect which suggests usage of this in hybrid development in cotton. Through effective implementation of hybrid cotton, India and China have achieved self-sufficiently in the production of cotton (Gao et al., 2016; Nachimuthu and Webb, 2017), but at present stage, research related to hybrid cotton development is at initial stage in Pakistan.

## Conclusion

It is concluded that forty diverse cotton genotypes have great genetic potential to breed cotton under water deficit condition. In the present study, suitable parents and their promising crosses for the improvement of the yield characteristics of cotton in water deficit conditions have been identified. Regarding the results of the principal component analysis (PCA), total variation exhibited by factors 1 and factor 2 were 55.55 and 41.95%, respectively. The overall, among the forty cotton genotypes AS-01 and CRS-456 on the basis of stress susceptibility index and FH-113 and FH-142 on the basis of seed cotton yield, performed better und water deficit conditions. In this regard, the cross-combinations VH-144 × S-12 and NIAB-111 × IR-3 have an effective result under water deficit conditions for most of the traits. SCA and dominance variances were positive and higher than GCA in magnitude under both normal and water deficit conditions. As all of the traits are being controlled by non-additive type of gene action, therefore, heterosis breeding is recommended for future breeding program for developing hybrids suitable for cultivation under water deficit areas of Pakistan. In case of development of cotton variety, crop selection must be delayed to latter generations until the fixation of segregating genes.

## Data availability statement

The original contributions presented in the study are included in the article/Supplementary material, further inquiries can be directed to the corresponding author.

## Author contributions

AU and AS performed the experiment and wrote the initial draft. HA, ANS, MA, and AU initiated and designed the research, collected the data, and wrote the final draft. MN, ANS, MA, AU, and HA analyzed the data. LW, MJ, NA, RG, and MH critically reviewed, validated the research, edited the manuscript, and helped in formatting. All authors contributed to the article and approved the submitted version.

## Funding

This work was financially supported by King Abdullah University of Science and Technology (KAUST), Thuwal, Saudi Arabia and Alexandria University, Alexandria, Egypt.

## Conflict of interest

The authors declare that the research was conducted in the absence of any commercial or financial relationships that could be construed as a potential conflict of interest.

## Publisher's note

All claims expressed in this article are solely those of the authors and do not necessarily represent those of their affiliated organizations, or those of the publisher, the editors and the reviewers. Any product that may be evaluated in this article, or claim that may be made by its manufacturer, is not guaranteed or endorsed by the publisher.
