# Supplementary material for: Genetic basis and principal component analysis in cotton (Gossypium hirsutum L.) grown under water deficit condition
Source: Front Plant Sci. 2022 Oct 6;13:981369. doi: 10.3389/fpls.2022.981369 (PMC9583382; doi:10.3389/fpls.2022.981369)
Supplement: Supplementary file 1 [file Data_Sheet_1.docx]

| Sr. No | Cultivar/Lines | Origin | Sr. No | Cultivar/Lines | Origin |
| --- | --- | --- | --- | --- | --- |
| 1 | AA-703 | Ali Akbar Seeds | 21 | IR-3 | NIBGE, Faisalabad |
| 2 | AA-802 | Ali Akbar Seeds | 22 | IR-3701 | NIBGE, Faisalabad |
| 3 | AS-01 | Exotic | 23 | IR-901 | NIBGE, Faisalabad |
| 4 | CRS-2007 | CRS, Multan | 24 | IUB-212 | IUB, Bahawalpur |
| 5 | CRS-456 | CRS, Multan | 25 | IUB-222 | IUB, Bahawalpur |
| 6 | FH-113 | CRI, Faisalabad | 26 | KZ-181 | Kanzo Seeds |
| 7 | FH-114 | CRI, Faisalabad | 27 | MG-6 | Exotic |
| 8 | FH-118 | CRI, Faisalabad | 28 | NIAB-111 | NIAB, Faisalabad |
| 9 | FH-142 | CRI, Faisalabad | 29 | NIAB-820 | NIAB, Faisalabad |
| 10 | FH-1000 | CRI, Faisalabad | 30 | MNH-888 | CRS, Multan |
| 11 | FH-169 | CRI, Faisalabad | 31 | MNH-886 | CRS, Multan |
| 12 | FH-170 | CRI, Faisalabad | 32 | NS-121 | Neelam Seeds |
| 13 | FH-171 | CRI, Faisalabad | 33 | NS-131 | Neelam Seeds |
| 14 | FH-172 | CRI, Faisalabad | 34 | S-12 | Sitara Seeds |
| 15 | FH-175 | CRI, Faisalabad | 35 | SB-149 | Exotic |
| 16 | CIM-707 | CCRI, Multan | 36 | VH-148 | CRS, Vehari |
| 17 | CIM-443 | CCRI, Multan | 37 | VH-144 | CRS, Vehari |
| 18 | CIM-240 | CCRI, Multan | 38 | VH-282 | CRS, Vehari |
| 19 | MNH-147 | CRS, Multan | 39 | VH-283 | CRS, Vehari |
| 20 | FH-941 | CRI, Faisalabad | 40 | VH-295 | CRS, Vehari |

**Table S1: List of 40 cotton accessions used in the current study**

CRS = Cotton Research Station, NIBGE = National Institute of Biotechnology and Genetic Engineering and CCRI = Central Cotton Research Institute.

**Fig. S1: Rainfall, relative humidity and average temperature from April to November during experiment-1**

**Fig. S2: Rainfall, relative humidity and average temperature from April to November during experiment-2**

(Data Source; Agromet Bulletin, Agriculture Meteorology Cell, Department of Crop Physiology, UAF, Pakistan)
